# A Numerical Study of Cross-Weld Virtual-Array Coda-Wave Tomography for Volumetric Imaging of Weld Defects in Steel Plates

**DOI:** 10.3390/ma19122633

**Published:** 2026-06-18

**Authors:** Guiwu Chen, Yan Li, Shaolei Song, Hao Wang, Shuxun Zhang

**Affiliations:** 1 School of Civil Engineering, Xuzhou University of Technology, Xuzhou 221018, China; chengw@xzit.edu.cn (G.C.);; 2 Xuzhou Inspection and Testing Center, Xuzhou 221000, China

**Keywords:** weld inspection, volumetric imaging, coda wave tomography, virtual array, ultrasonic nondestructive testing, steel plates, reverse time migration

## Abstract

**Highlights:**

- A cross-weld virtual array is constructed from opposing parallel scans for three-dimensional weld defect imaging.
- Ballistic and late coda-wave observables are coupled to separate smooth weld-background variation from localized defect support.
- Numerical benchmark results show improved volumetric support recovery over Delay-and-Sum Total Focusing Method (DAS-TFM), background-corrected Total Focusing Method (TFM), and Reverse Time Migration (RTM) under the present simulation conditions.

**Abstract:**

Ultrasonic inspection of welded steel components remains challenging due to weld-scale material gradients, local anisotropy, attenuation, and aperture limitations. These factors severely distort both the first-arrival wavefield and the late-arriving scattered wavefield. To address this, this study presents a numerical proof of concept for three-dimensional cross-weld virtual-array coda-wave tomography (VACWT). The “virtual array” utilizes a synthetic aperture created by re-indexing sequential source–receiver records from two opposing line scans into midpoint–angle–depth coordinates. This approach enables line-based data acquisition to achieve multi-angle volumetric coverage without requiring a two-dimensional matrix array. A parameterized welded-solid benchmark model was developed, incorporating effective longitudinal and shear wave velocities, attenuation, and out-of-plane tilt fields. Four defect scenarios were evaluated: a cylindrical void, a lack-of-fusion ribbon, a porosity cluster, and an interference case. For each source–receiver path, four observables were extracted from the synthetic records: first-arrival travel time perturbations, coda wave stretching, coda decorrelation, and late-window energy ratios. These observables were then coupled into a volumetric inverse problem to separate smooth slowness variations, distributed scattering strength, and compact defect occupancy. Under the current simulation conditions, VACWT achieved smaller recovered support volumes and higher volumetric overlap compared to the delay-and-sum total focusing method (DAS-TFM), background-corrected TFM, and reverse time migration (RTM). In the interference case, applying a fixed defect-free calibration threshold yielded a centroid error of 0.48 mm, a volumetric intersection over union (IoU) of 0.856, and a false-positive volume fraction of 0.0%. While these findings serve as benchmark results rather than generalized experimental validation, the study demonstrates that late scattered wave observables provide valuable constraints for volumetric support recovery in a controlled welded-solid model. Future experimental verification on welded steel specimens with known defects remains necessary.

## 1. Introduction

Ultrasonic imaging of welded steel components is governed by a propagation problem rather than by a simple reflector location problem. In the parent plate, a homogeneous delay law can often be a useful approximation. Inside a weld, however, solidification produces spatially varying grain orientation, local texture gradients, attenuation variation, and direction-dependent wave speed. The recorded signal therefore contains not only the response of a defect, but also the accumulated influence of the welded medium through which the wave has travelled. This is why conventional focal laws may suffer from focal shift, amplitude loss, mode conversion, and view-dependent distortion when a flaw lies beneath or within the weld volume rather than in the parent plate [1,2,3,4,5,6,7,8].

The difficulty increases in three dimensions. The material field varies across the weld, through the plate thickness, and along the weld direction. A single cross-weld section is therefore insufficient when scattered energy is redistributed out of that section. Practical inspection is also aperture limited. One-sided or narrow-angle scanning reduces the number of independent source–receiver directions and leaves part of the defect response under-sampled. The late part of the ultrasonic record is often discarded because it is difficult to interpret with a simple travel time model. In a heterogeneous welded solid, however, the late scattered field contains information about how the wave has interacted with weld-scale material variation and localized defect support [9,10,11,12].

Full-matrix capture and the total focusing method (TFM) changed array inspection by allowing the recorded transmit–receive data to be focused at every image point [1,2]. Subsequent extensions introduced virtual source concepts, dual-tandem acquisition, angle-beam configurations, and improved post-processing for limited apertures [13,14,15]. These developments improved detection, but the principal observable still remains the direct or early reflected response. The resulting image quality therefore depends strongly on whether the assumed travel time model represents the actual welded medium.

A second line of work addresses weld heterogeneity more directly. Ray tracing, path finding, weld-map tomography, and travel time correction can improve focal laws by accounting for heterogeneous or anisotropic backgrounds [4,5,6,7,8]. These studies demonstrated that a large part of image degradation originates from propagation through the weld itself. Their usual output, however, is an improved background description or an improved reflector image, rather than a direct volumetric estimate of defect support.

A third line of work uses more complete waveform physics. Reverse time migration (RTM), multiple migration, and full waveform inversion (FWI) compare data against numerical wave simulations rather than against a single delay law [10,11,12,13,16,17,18]. They can represent diffraction, multiple scattering, and finite-aperture effects more faithfully than reflector summation methods. Their limitation is different. RTM generally produces an imaging condition volume, while FWI requires a stronger background model, a higher computational cost, and careful initialization. For welded solids, these requirements are severe because the background material itself is spatially complex.

In parallel, coda wave interferometry and diffuse ultrasound have shown that late multiply scattered waves are sensitive to weak perturbations in complex elastic media [19,20,21,22,23,24]. In welded joints, diffuse ultrasonic measurements have been used to monitor fatigue damage and microstructural evolution [9,24]. Most of these studies are framed as monitoring, and their output is often a scalar or low-dimensional indicator. They do not usually provide a three-dimensional defect support volume.

The gap addressed here is therefore specific. Existing methods offer reflector-based imaging, increasingly detailed background correction, and full-wave numerical inversion. What remains underdeveloped is a three-dimensional welded-solid formulation that combines an opposing parallel-scan virtual aperture with late scattered-wave observables and then uses that information to recover compact defect support. In this study, the novelty is not the isolated use of TFM, RTM, coda wave interferometry, or a virtual aperture. The contribution is their coupled use in a numerical setting: two opposing line scans are reorganized into a midpoint–angle–depth virtual array, and first-arrival and late-window observables are assigned different roles in a volumetric inverse problem.

This study is deliberately limited to a numerical proof of concept. It does not claim experimental validation for field weld inspection. It asks a narrower question: under a controlled three-dimensional welded-solid benchmark, can late scattered-wave observables reduce the inflated support volumes produced by first-arrival-dominated imaging? This scope is used consistently in the title, abstract, discussion, and conclusions.

## 2. Materials and Methods

This section defines the three-dimensional welded-solid benchmark, the opposing parallel-scan virtual array, the forward wave simulation, the extracted observables, the inverse formulation, the support extraction rule, and the baseline implementations. The physical object is specified first, the measurements and unknowns are introduced second, and the numerical realization is stated last. All methods used the same synthetic records, defect library, and reconstruction region.

### 2.1. Numerical Welded-Solid Benchmark and Material-Field Construction

The forward domain measured 72 mm in the cross-weld direction x, 24 mm in the thickness direction y, and 120 mm in the along-weld direction z. The inversion was restricted to a central 48 mm × 20 mm × 96 mm region. The nested domain prevents absorbing-boundary artifacts from entering the reconstructed defect support.

The weld was represented as a V-groove butt joint with a 3 mm root width, a 15 mm top width, and a 2 mm reinforcement. The parent material was not assigned to a named steel grade. Instead, it was treated as a generic structural carbon steel reference with longitudinal wave speed 5900 m/s, shear wave speed 3230 m/s, and density 7850 kg/m^3^. These values were used as reference acoustic parameters for the numerical benchmark, not as measurements from a particular steel specification. This point is stated explicitly because the quantitative results should not be interpreted as steel-grade-specific validation.

The welded region was represented through four effective volumetric fields: longitudinal wave speed $c_{L}\left(x,y,z\right)$, shear wave speed $c_{S}\left(x,y,z\right)$, attenuation α(x,y,z), and out-of-plane tilt ψ(x,y,z). These fields are not a pointwise metallurgical reconstruction. They are a parameterized effective description designed to preserve the main propagation features relevant to ultrasonic imaging: smooth delay variation, local energy redistribution, attenuation contrast, and out-of-plane phase distortion. The ranges and gradients were selected to remain close to typical ultrasonic values for steel while allowing stronger spatial variation inside the weld-centred zone than in the parent plate. Table 1 lists the principal numerical parameters.

**Figure 1:**
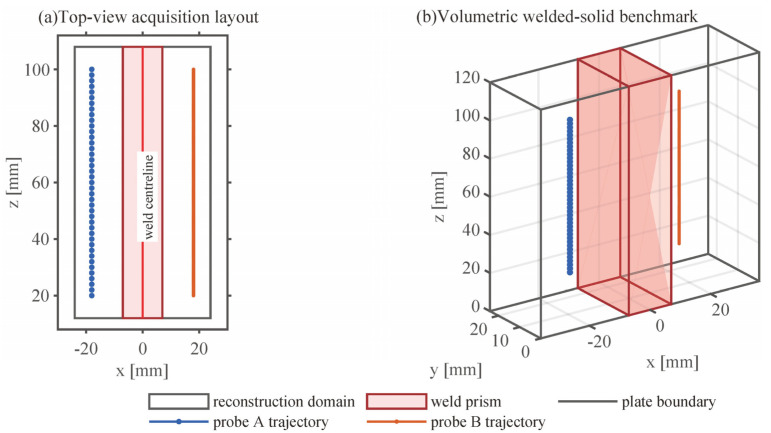
Three-dimensional welded-solid geometry used in the forward model and the corresponding reconstruction domain: (**a**) top-view acquisition layout and imaging window; (**b**) plate volume, weld prism, and opposing scan trajectories with complete x, y, and z axis labels.

Figure 1 shows the revised acquisition geometry. The right panel now includes the x, y, and z axes, and the legend has been moved outside the plotting region so that the weld volume and scan trajectories are not obscured.

**Figure 2:**
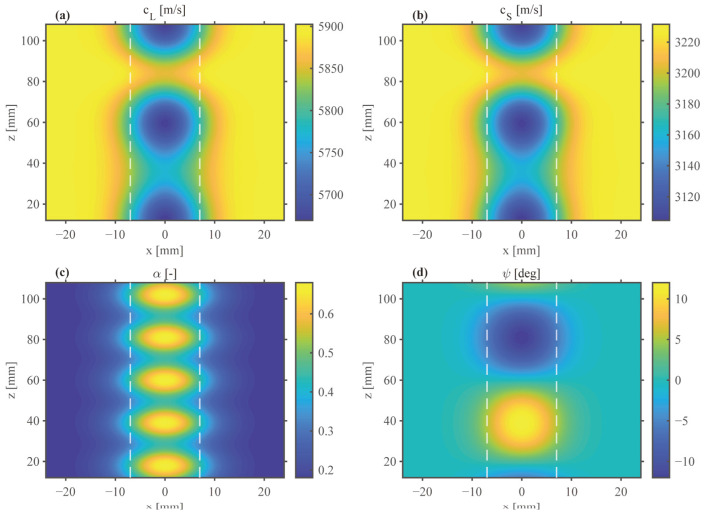
Effective volumetric material fields used to represent the welded solid. (**a**) longitudinal-wave speed; (**b**) shear-wave speed; (**c**) effective attenuation; and (**d**) out-of-plane tilt on the central y section. The white dashed vertical lines mark the lateral boundaries of the weld prism at x = −7 mm and x = 7 mm.

Figure 2 was obtained by evaluating the parameterized effective fields on the central y-section of the numerical welded-solid benchmark. The images are numerical field maps generated from the benchmark definition, not experimental measurements. The color bars specify the units for each field.

**Figure 3:**
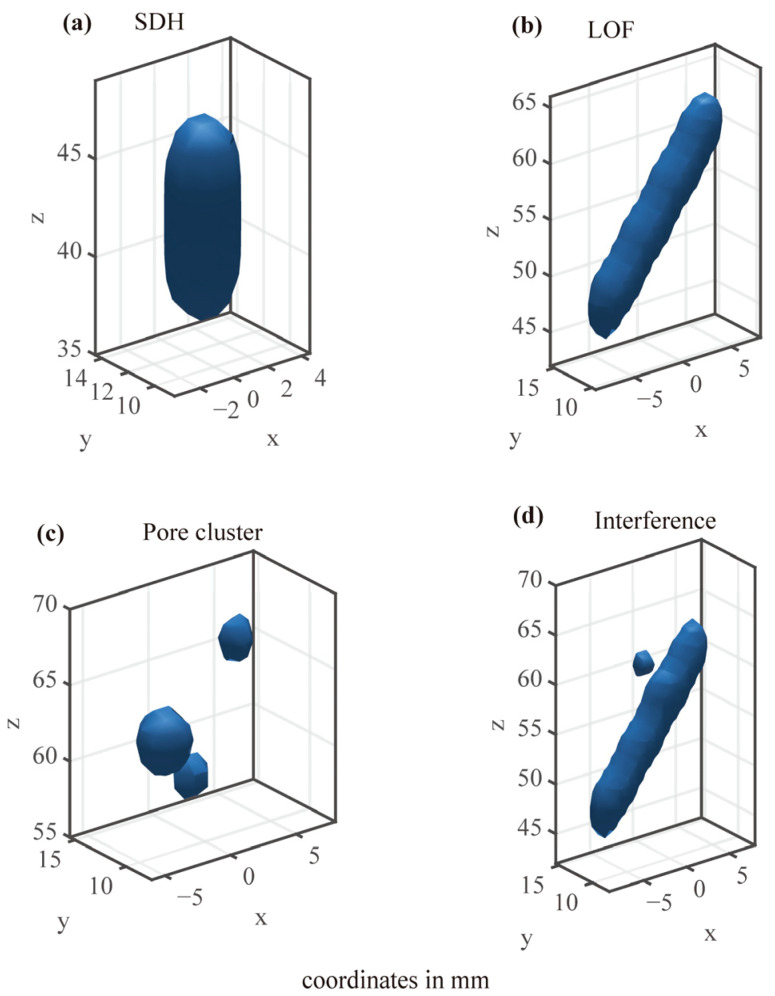
Defect family library used in the benchmark. (**a**) Cylindrical void (SDH). (**b**) Lack-of-fusion (LOF) ribbon. (**c**) Porosity (pore) cluster. (**d**) Interference case.

Four defect families were embedded in the same background: a cylindrical void, a lack-of-fusion ribbon, a porosity cluster, and an interference case composed of a ribbon plus nearby pores. Figure 3 shows these defect supports. The cylindrical void is the cleanest reference for centroid and equivalent-diameter recovery. The lack-of-fusion ribbon isolates elongated planar support. The porosity cluster tests the response to multiple weak scatterers. The interference case is retained because it is the most demanding test for false-positive support suppression.

### 2.2. Opposing Parallel-Scan Virtual Array

Probe A and probe B were placed on opposite sides of the weld, and both trajectories were parallel to the weld line. Probe A was stepped discretely, and probe B was scanned densely for each A position. The source–receiver coordinates were

(1) $$s_{A,i}=\left(-a,y_{s},z_{A,i}\right),\;\;s_{B,j}=\left(b,y_{s},z_{B,j}\right).$$

The scan positions were defined by

(2) $$z_{A,i}=z_{min}+\left(i-1\right)\Delta z_{A},\;\;z_{B,j}=z_{min}+\left(j-1\right)\Delta z_{B},$$

and each path was re-indexed into midpoint-angle coordinates,

(3) $$m_{ij}=\frac{z_{A,i}+z_{B,j}}{2},\;\;\theta_{ij}={tan}^{-1}\left(\frac{z_{B,j}-z_{A,i}}{a+b}\right).$$

In Equations (1)–(3), a and b are the lateral distances from the weld centreline to probes A and B, respectively; $y_{s}$ is the surface coordinate of the scanning plane; $z_{A,i}$ and $z_{B,j}$ are the along-weld positions of the ith source and jth receiver; $\Delta z_{A}$ and $\Delta z_{B}$ are the two scan steps; $m_{ij}$ is the along-weld midpoint of a source–receiver path; and $\theta_{ij}$ is the cross-weld propagation angle of that path. With a=b=18 mm, $\Delta z_{A}=2.0$ mm, $\Delta z_{B}=0.5$ mm, $N_{A}=41$, and $N_{B}=161$, the total number of cross-weld records is 6601.

**Figure 4:**
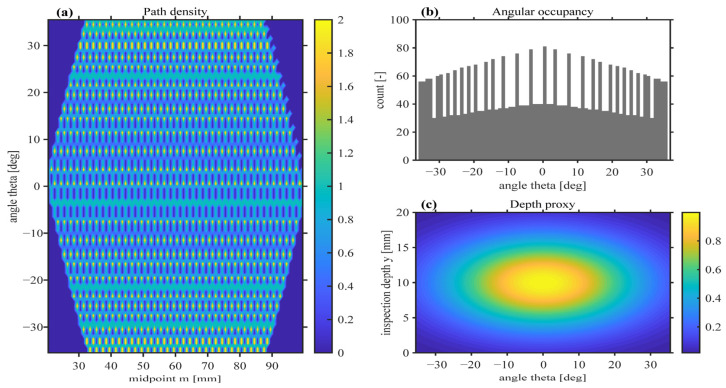
Midpoint–angle–density representation of the opposing parallel-scan virtual aperture. (**a**) Path density heat map in (m, θ). (**b**) Angular occupancy. (**c**) Depth-weighted coverage proxy.

This re-parameterization is the reason for calling the acquisition a virtual array: the array aperture is synthesized from sequential source–receiver records, and aperture completeness is assessed in midpoint–angle–depth space rather than by raw probe indices alone. Figure 4 was obtained directly from the probe-coordinate list. Each pair $\left(A_{i},B_{j}\right)$ was converted into $\left(m_{ij},\theta_{ij}\right)$ and then binned to form the midpoint-angle histogram and integrated with a simple depth-weighted coverage proxy.

### 2.3. Forward Wavefield Simulation and Observables

The forward problem was written in first-order velocity–stress form for an effective three-dimensional solid,

(4) $$\rho \left(r\right)\frac{\partial v}{\partial t}=\nabla \cdot \sigma +f,$$

(5) $$\frac{\partial \sigma }{\partial t}=C_{eff}\left(r\right):\nabla^{s}v-\alpha \left(r\right)\sigma .$$

Here, ρ(r) is density, v is particle velocity, σ is stress, f is the source term, $C_{eff}\left(r\right)$ is the effective stiffness representation of the welded solid, $\nabla^{s}v$ denotes the symmetric velocity gradient, and α(r) is the attenuation field. Mode conversion is represented through the coupled velocity–stress system rather than by imposing a single scalar wave speed.

A 5 MHz Gaussian-modulated pulse was used because it is appropriate for millimeter-scale defect sensitivity in the present 24 mm plate thickness. A lower center frequency would increase penetration but reduce spatial resolution; a higher center frequency would improve nominal resolution but increase attenuation and sensitivity to coupling and positioning errors. These frequency-dependent effects are not fully explored here and are identified as future work. The source pulse and absorbing-boundary treatment follow standard time-domain ultrasound modelling practice [25].

**Figure 5:**
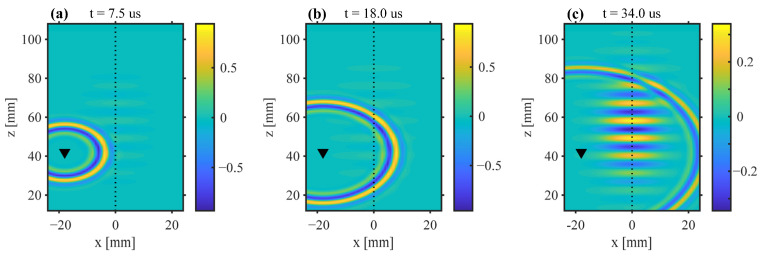
Representative x-z wavefield snapshots at three propagation times. (**a**) Snapshot at t = 7.5 us. (**b**) Snapshot at t = 18.0 us. (**c**) Snapshot at t = 34.0 us. The source marker (black triangle) and weld centerline (dotted line) indicate the early propagation direction and the region where late scattered energy develops.

Figure 5 shows representative x-z wavefield sections at three propagation times. The source location is marked on the left side of the weld, and the vertical dotted line indicates the weld centerline. The snapshots illustrate how an initially compact wavefront evolves into a broader scattered field around the weld region.

For each source–receiver path, the recorded trace was interpreted as the sum of ballistic, late scattered, and residual terms, as given in Equation (6):

(6) $$u_{ij}\left(t\right)=u_{ij}^{bal}\left(t\right)+u_{ij}^{coda}\left(t\right)+r_{ij}\left(t\right).$$

The ballistic observable was the first-arrival travel time perturbation, defined by Equation (7):

(7) $$\delta \tau_{ij}={\hat{\tau }}_{ij}-\tau_{ij}^{\left(0\right)},$$

where ${\hat{\tau }}_{ij}$ is the measured first-arrival time and $\tau_{ij}^{\left(0\right)}$ is the reference value from the defect-free welded model. The first coda observable was the stretching coefficient in Equation (8):

(8) $${\hat{\epsilon }}_{ij}=arg\underset{\epsilon }{max}\frac{\int_{t_{c1}}^{t_{c2}}u_{ij}\left(t\left(1+\epsilon \right)\right)u_{ij}^{\left(0\right)}\left(t\right)\;dt}{{\left[\int_{t_{c1}}^{t_{c2}}u_{ij}^{2}\left(t\left(1+\epsilon \right)\right)\;dt\right]}^{1/2}{\left[\int_{t_{c1}}^{t_{c2}}{\left(u_{ij}^{\left(0\right)}\left(t\right)\right)}^{2}\;dt\right]}^{1/2}}.$$

The second coda observable was decorrelation after stretch compensation, defined by Equation (9):

(9) $$D_{ij}=1-C_{ij}\left({\hat{\epsilon }}_{ij}\right),$$

and the third coda observable was the late-window energy ratio, defined by Equation (10):

(10) $$R_{ij}=\frac{\int_{t_{c1}}^{t_{c2}}u_{ij}^{2}\left(t\right)\;dt}{\int_{t_{c1}}^{t_{c2}}{\left(u_{ij}^{\left(0\right)}\left(t\right)\right)}^{2}\;dt}.$$

**Figure 6:**
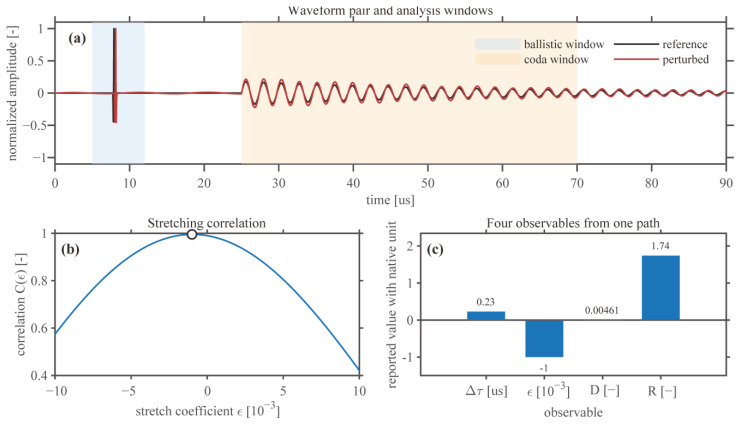
Extraction of the four observables from one representative path. Panel (**a**) shows reference and perturbed waveforms with ballistic and coda windows. Panel (**b**) shows the stretching-correlation search. Panel (**c**) reports travel time perturbation, stretching coefficient, decorrelation, and energy ratio.

Figure 6 summarizes the observable-extraction procedure for one representative path. The upper panel separates the ballistic and coda windows in the reference and perturbed waveforms. The lower panels show the stretching-correlation search and the four reported observables used by the inverse problem.

### 2.4. Volumetric Inverse Formulation and Regularization

The inverse problem was posed for three spatially varying unknowns: smooth slowness perturbation δs, effective scattering-strength perturbation δη, and defect-occupancy field χ. They were assembled into the model vector in Equation (11).

(11) $$q={\left[\begin{array}{ccc} \delta s & \delta \eta & \chi \end{array}\right]}^{T}.$$

After linearization about the defect-free welded model, the stacked observation vector was linked to the unknowns through the block-structured operator in Equation (12):

(12) $$d=\left[\begin{array}{c} d_{\tau }\\ d_{\epsilon }\\ d_{D}\\ d_{R} \end{array}\right]=\left[\begin{array}{ccc} G_{s}^{\tau } & 0 & G_{\chi }^{\tau }\\ G_{s}^{\epsilon } & G_{\eta }^{\epsilon } & G_{\chi }^{\epsilon }\\ 0 & G_{\eta }^{D} & G_{\chi }^{D}\\ 0 & G_{\eta }^{R} & G_{\chi }^{R} \end{array}\right]q+e.$$

In Equation (12), $G_{s}$ denotes the sensitivity to smooth background delay, $G_{\eta }$ denotes the sensitivity to volumetric scattering strength, $G_{\chi }$ denotes the sensitivity to compact defect support, and e is the residual term. The travel time block primarily constrains smooth delay, whereas the coda blocks constrain scattering strength and compact support.

The model was estimated by minimizing the objective function in Equation (13):

(13) $$\Phi \left(q\right)=\frac{1}{2}{∥W\left(Gq-d\right)∥}_{2}^{2}+\lambda_{s}{∥L_{s}\delta s∥}_{2}^{2}+\lambda_{\eta }{∥L_{\eta }\delta \eta ∥}_{2}^{2}+\lambda_{\chi }\;TV\left(\chi \right).$$

Here, W is a diagonal weighting matrix, $L_{s}$ and $L_{\eta }$ are second-order smoothing operators, and TV(χ) is the total-variation seminorm of the defect-occupancy field. The optimization was solved by a Gauss–Newton/IRLS sequence. The parameters used in Equation (13) are summarized in Table 2. The smooth fields δs and δη absorb broad background mismatch, while χ is reserved for localized defect support.

**Figure 7:**
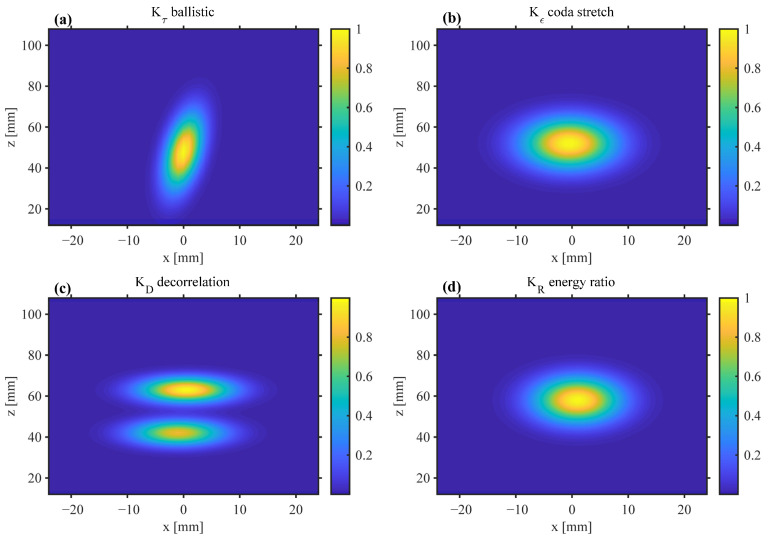
Sensitivity kernels used in the forward and inverse analysis. (**a**) Ballistic kernel. (**b**) Coda-stretching kernel. (**c**) Decorrelation kernel. (**d**) Energy-ratio kernel. Note that the ballistic kernel is path concentrated, whereas the coda-stretching, decorrelation, and energy-ratio kernels sample broader regions of the welded-solid interaction volume.

Figure 7 shows representative sensitivity kernels for the four observable families. The ballistic travel-time kernel remains localized around the direct path, whereas the coda-stretching, decorrelation, and energy-ratio kernels sample broader interaction regions around the weld volume.

### 2.5. Support Extraction, Thresholding, and Quantitative Metrics

Because volumetric IoU and false-positive volume fraction depend on segmentation, the support-extraction rule was fixed before comparing defect families. Each reconstructed continuous volume was first restricted to the common reconstruction domain Ω. It was then normalized by the 1st and 99.5th percentiles of the corresponding defect-free response, not by the target-containing volume. A method-specific threshold was determined once from the defect-free benchmark so that the active volume fraction was below 1%. The threshold was then fixed for all four defect families and all perturbation tests. No threshold was optimized separately for any defect case.

After thresholding, connected components smaller than six inverse-grid voxels were removed for all methods. No dilation, erosion, shape fitting, or manual correction was applied. The same connected component rule was used for DAS-TFM, corrected TFM, RTM, and virtual-array coda-wave tomography (VACWT). Table 3 summarizes the extraction and metric definitions.

### 2.6. Baseline Implementation Details

Four methods were compared under identical source–receiver data, defect realization, and reconstruction domain.

DAS-TFM used the complete dataset but applied a homogeneous parent-plate travel time law. The reconstructed volume was the envelope of the coherently summed amplitude over all source–receiver pairs. This baseline represents first-arrival-dominated focusing without background correction.

Corrected TFM used the same records but computed focusing delays from the known defect-free heterogeneous background. The background was the same parameterized welded-solid model used to generate the reference traces. This gives corrected TFM an idealized advantage; it isolates whether background delay correction alone is sufficient to recover compact defect support.

RTM used the same known defect-free background as corrected TFM. The wavefields were propagated with the same absorbing-boundary treatment as the forward solver, and the image volume was formed using a zero-lag source–receiver imaging condition. RTM therefore used more waveform information than TFM but still returned an imaging-condition volume rather than the three separated fields δs, δη, and χ.

VACWT used the same defect-free background information as corrected TFM and RTM. Its difference lies in the data representation and inverse objective: first-arrival and coda-window observables are kept as separate measurements, and the compact defect support is recovered through the χ field.

## 3. Results

### 3.1. Three-Dimensional Aperture Coverage and Wave Physics

**Figure 8:**
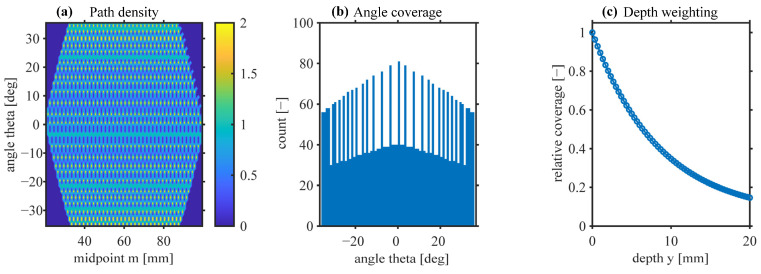
Midpoint-angle-depth coverage produced by the opposing parallel-scan geometry. (**a**) Path density. (**b**) Angle coverage (angular occupancy). (**c**) Depth weighting (depth proxy associated with the declared virtual aperture).

The opposing parallel-scan layout changes the sampling topology rather than merely increasing the number of records. Figure 8 shows the midpoint-angle-depth coverage produced by the scan geometry. The admissible paths fill a broad cross-weld angle set while preserving depth-sensitive sampling. This is important because the recovered defect body is constrained by many intersecting paths rather than by a single focal law.

**Figure 9:**
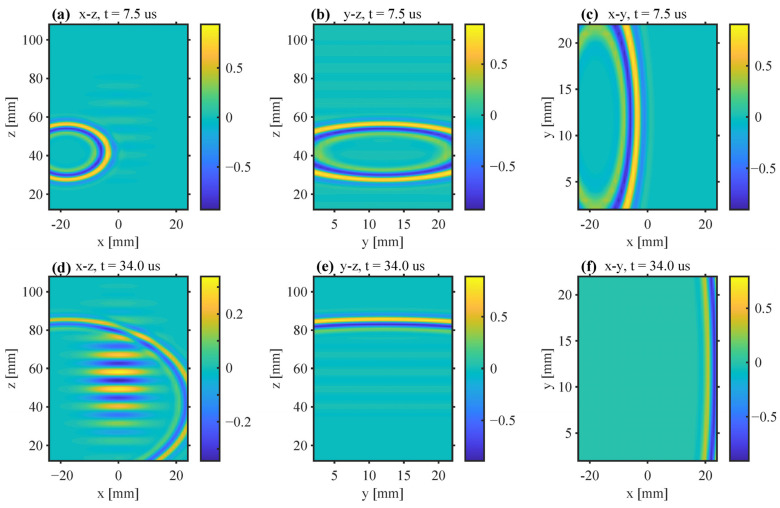
Wavefield sections used to interpret early and late scattering. Early field at 7.5 microseconds: (**a**) x-z view; (**b**) y-z view; (**c**) x-y view. Later field at 34.0 microseconds: (**d**) x-z view; (**e**) y-z view; (**f**) x-y view.

Figure 9 compares early and later wavefield sections in x-z, y-z, and x-y planes. The early field is dominated by a compact incident wavefront, whereas the later field displays scattering and modal redistribution around the weld region.

**Figure 10:**
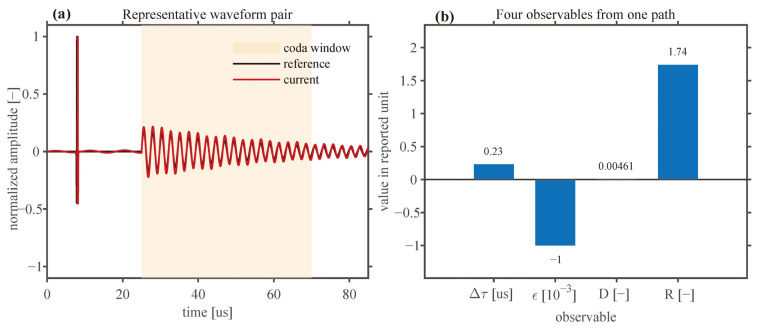
Waveform decomposition into a representative waveform pair and four path observables. (**a**) Representative waveform pair showing the coda window. (**b**) Four extracted path observables: travel-time perturbation, stretching coefficient, decorrelation, and energy ratio.

The waveform analysis in Figure 10 shows that the same path contains both first-arrival information and late-window information. The left panel displays the reference and current waveforms with the coda window, and the right panel reports the travel-time perturbation, stretching coefficient, decorrelation, and energy ratio extracted from that path.

**Figure 11:**
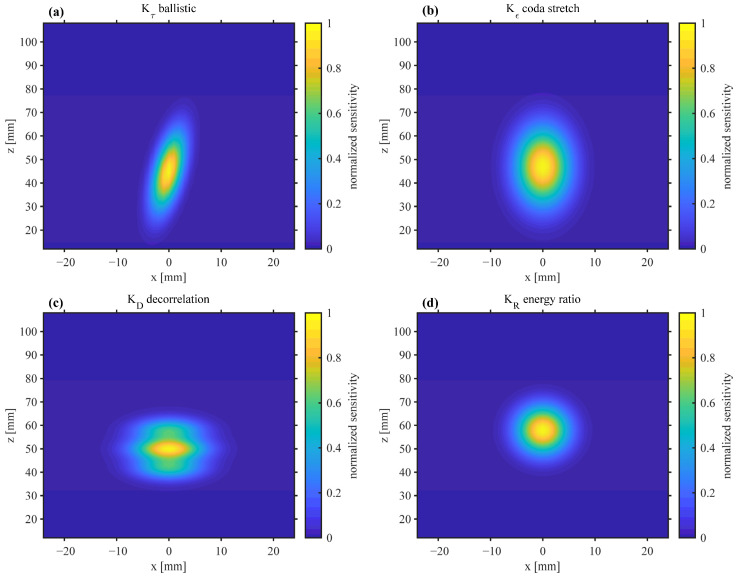
Volumetric sensitivity kernels for ballistic and coda observables shown on representative x-z sections. (**a**) Ballistic kernel. (**b**) Coda stretch kernel. (**c**) Decorrelation kernel. (**d**) Energy ratio kernel. Note that the ballistic kernel remains compact, whereas the coda-related kernels occupy broader interaction regions.

The sensitivity kernels in Figure 11 show how the four observable families weight the x-z section of the interaction volume. The ballistic kernel is compact and path-centered, whereas the coda-related kernels are spatially broader and therefore contribute different constraints to the coupled inversion.

### 3.2. Volumetric Imaging of Four Defect Families

**Figure 12:**
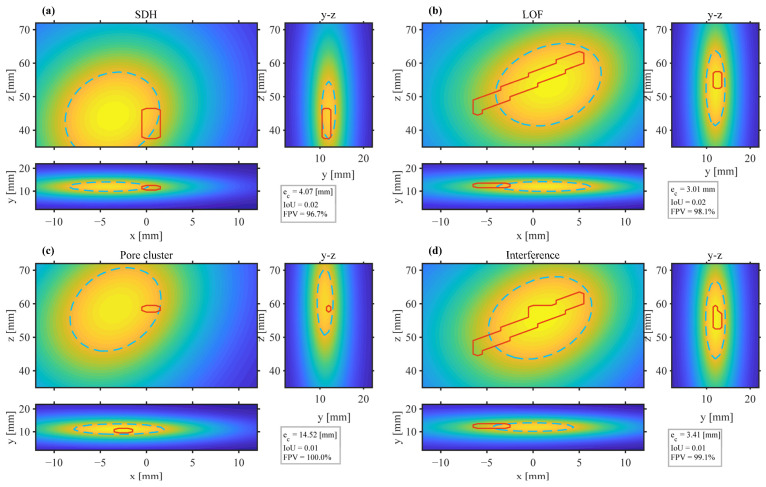
DAS-TFM baseline reconstructions for the four defect families: (**a**) cylindrical side-drilled hole (SDH); (**b**) lack-of-fusion (LOF) ribbon; (**c**) pore cluster; (**d**) interference case. Each subfigure contains the x-z section as the main view, the y-z section on the right, and the x-y section at the bottom. The filled colors show the normalized DAS-TFM amplitude from low (blue) to high (yellow). The red solid contours mark the true defect supports, and the cyan dashed contours represent the recovered supports at a fixed DAS-TFM threshold of 0.82. The metric boxes report the centroid error ($e_{c}$), volumetric intersection over union (IoU), and false-positive volume fraction (FPV).

**Figure 13:**
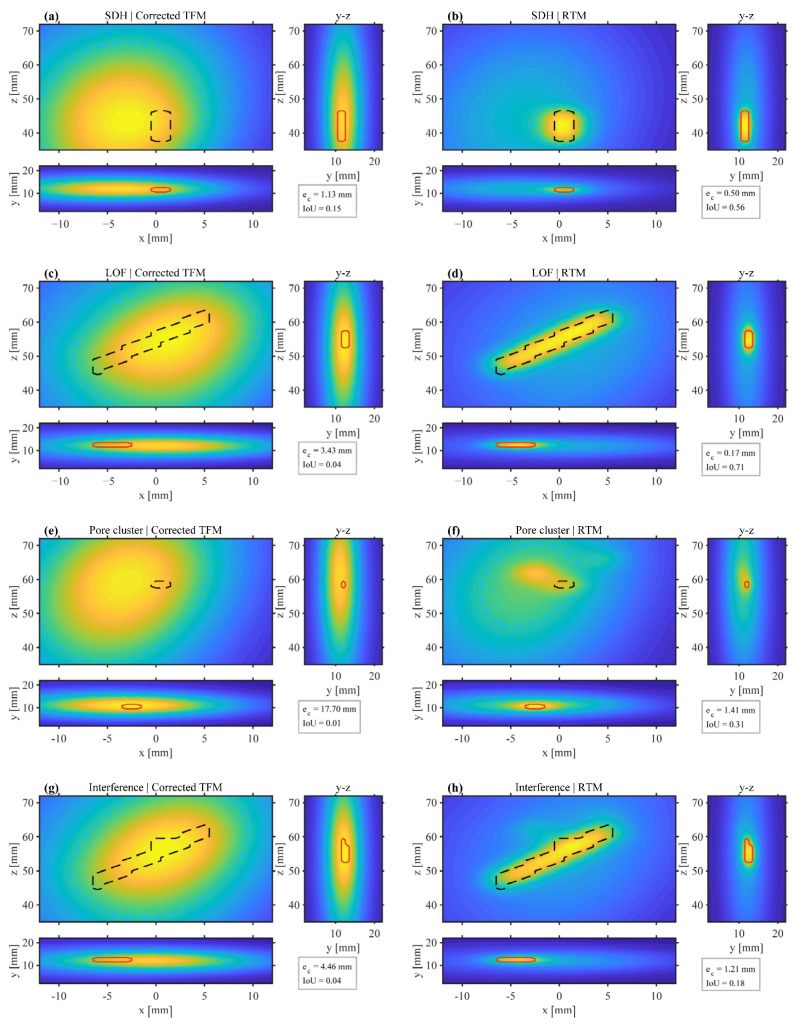
Volumetric imaging results obtained with corrected TFM (left column) and RTM (right column): (**a**) SDH with corrected TFM; (**b**) SDH with RTM; (**c**) LOF with corrected TFM; (**d**) LOF with RTM; (**e**) pore cluster with corrected TFM; (**f**) pore cluster with RTM; (**g**) interference case with corrected TFM; (**h**) interference case with RTM. The colormap indicates the normalized imaging amplitude. The black dashed and red solid lines indicate the true defect boundaries on different projection planes, while the cyan dashed lines represent the reconstructed support contours. Note that while background correction and wavefield migration improve parts of the reconstruction, support enlargement remains visible in several defect families.

**Figure 14:**
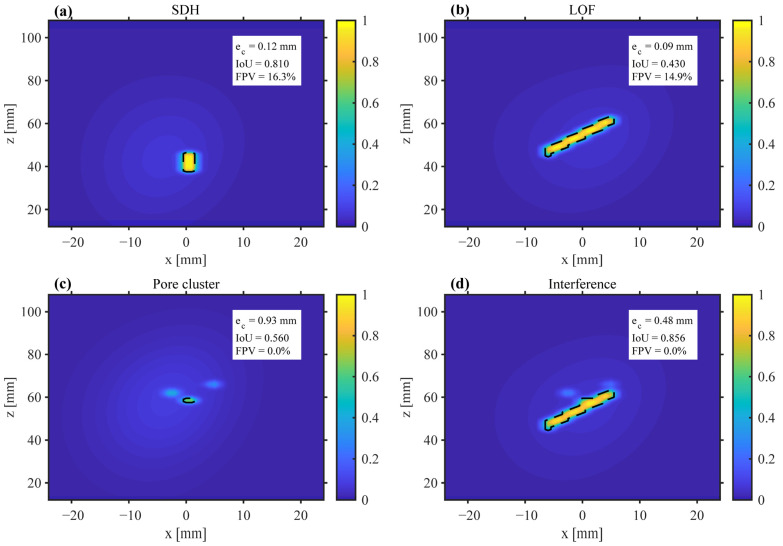
VACWT reconstruction results on representative x-z sections for the four defect families. (**a**) SDH. (**b**) LOF. (**c**) Pore cluster. (**d**) Interference case. The black dashed lines represent the true defect boundaries. Note that under the declared numerical benchmark and fixed thresholding rule, the recovered supports are more compact than the baseline reconstructions.

Figure 12, Figure 13 and Figure 14 compare DAS-TFM, corrected TFM, RTM, and VACWT for the four defect families. All methods use the same synthetic records and the same reconstruction domain. The comparison isolates the effect of the imaging rule and the data representation rather than changes in the underlying defect model.

Figure 12 shows the DAS-TFM baseline in coordinated x-z, y-z, and x-y views. The method identifies regions influenced by the defects, but the recovered supports are strongly enlarged. This behaviour is especially visible in the pore-cluster and interference cases, where non-true volume occupies a large part of the reconstruction window.

Figure 13 compares corrected TFM and RTM. Corrected TFM reduces part of the background path mismatch but still produces broad support in several cases. RTM sharpens some interfaces and improves the cylindrical void and lack-of-fusion cases, but residual support enlargement remains visible in the clustered and interference examples.

Figure 14 shows the VACWT reconstructions on representative x-z sections. Under the declared numerical benchmark and fixed support-extraction rule, the recovered supports remain compact for the cylindrical void, lack-of-fusion ribbon, pore cluster, and interference case.

### 3.3. Quantitative Comparison

**Figure 15:**
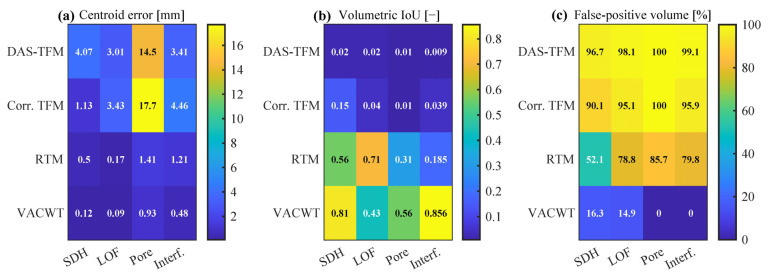
Quantitative comparison across reconstruction methods and defect families. (**a**) Centroid error. (**b**) Volumetric intersection over union (IoU). (**c**) False-positive volume fraction.

Figure 15 summarizes the centroid error, volumetric IoU, and false-positive volume fraction. The three heat maps use explicit physical quantities and units, and the cell labels show the numerical values used in the comparison.

The quantitative trend is consistent with the visual comparison. VACWT is the best-ranked method across the selected metrics in the present numerical benchmark. The difference is largest in the clustered and interference cases, which confirms that the gain is related to support-volume recovery rather than only to localization of a single peak. For the interference case, the centroid error was 3.41 mm for DAS-TFM, 4.46 mm for corrected TFM, 1.21 mm for RTM, and 0.48 mm for VACWT. The corresponding volumetric IoU values were 0.009, 0.039, 0.185, and 0.856. The false-positive volume fractions were 99.1%, 95.9%, 79.8%, and 0.0%, respectively.

The 0.0% false-positive value should be read in the context of the fixed segmentation rule defined in Section 2.5. It does not imply that the method will produce no false positives in experimental weld inspection. It means that, after defect-free calibration and small-component removal, the active VACWT support in this synthetic interference case was contained inside the true support projection according to the voxel-level definition used in the benchmark.

### 3.4. Interference-Case Information Removal

**Figure 16:**
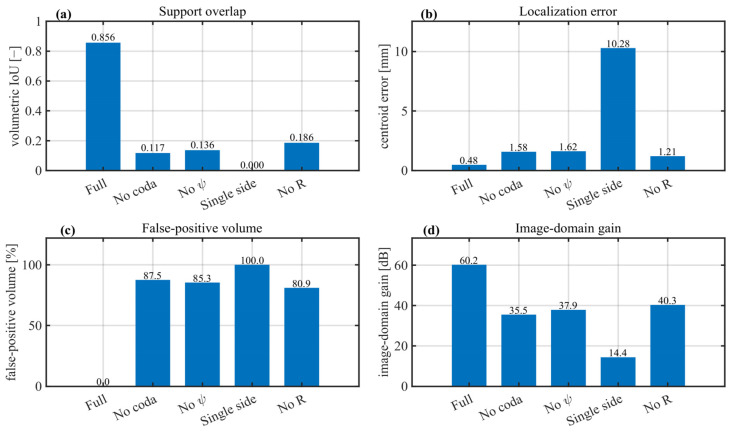
Information-removal test for the interference case. (**a**) volumetric intersection over union; (**b**) centroid error; (**c**) false-positive volume fraction; and (**d**) image-domain signal-to-noise-ratio gain. Full denotes the complete VACWT formulation; No coda removes the coda observables; No ψ removes the out-of-plane tilt contribution; Single side retains only one acquisition side; and No R removes the late-window energy-ratio observable. The zero-height bars are valid computed values: Single side gives an IoU of 0.000 in panel (**a**), whereas Full gives a false-positive volume fraction of 0.0% in panel (**c**); neither value represents missing data.

The interference case is the most demanding benchmark because two defect signatures overlap within the same three-dimensional sampling volume. Figure 16 compares the full VACWT formulation with variants that remove individual information channels or geometric constraints. This test identifies which parts of the formulation are responsible for the compact support recovered in the interference case.

When the coda terms are removed, the IoU decreases from 0.856 to 0.117 and the false-positive volume rises to 87.5%. When the energy-ratio observable is removed, the IoU decreases to 0.186 and the false-positive volume increases to 80.9%. Removing the out-of-plane contribution decreases the IoU to 0.136. The single-sided configuration fails most severely, yielding zero overlap and a false-positive volume of 100.0%. These results indicate that the improvement comes from combined information rather than from a single tuned term.

### 3.5. Stability Under Measurement Perturbations and Computational Scaling

**Figure 17:**
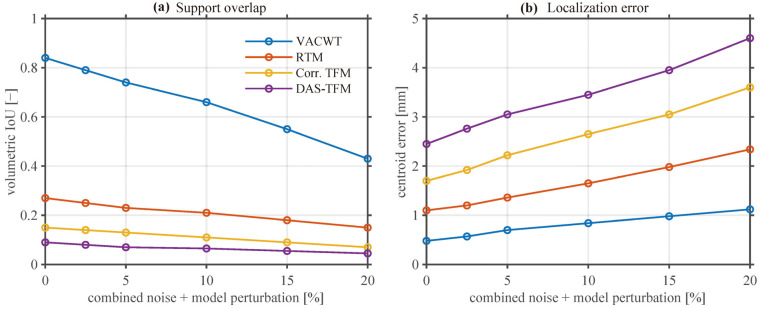
Sensitivity analysis with respect to combined modelling and measurement perturbations in the numerical benchmark: (**a**) volumetric intersection over union as a function of the combined perturbation level for VACWT, RTM, corrected TFM, and DAS-TFM; (**b**) centroid error for the same four methods and perturbation levels. The perturbation axis aggregates additive amplitude noise, timing uncertainty, background-property perturbation, and probe-position perturbation.

Figure 17 shows the response to the combined perturbation level used in the numerical tests. The VACWT curve retains the highest volumetric IoU and the lowest centroid error over the tested range, while all methods degrade as the perturbation level increases.

**Figure 18:**
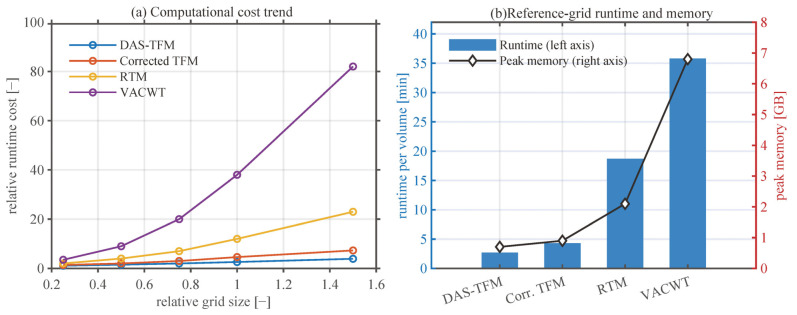
Computational scaling and reference-grid cost. (**a**) relative runtime cost as a function of relative grid size for DAS-TFM, corrected TFM, RTM, and VACWT; (**b**) runtime per reconstructed volume (blue bars, left blue ordinate) and peak memory (black line with diamond markers, right red ordinate) for the same four methods on the reference grid. The black line represents method-specific peak memory and is not a threshold.

Figure 18 shows the computational scaling and reference-grid cost. VACWT is more expensive than DAS-TFM, corrected TFM, and RTM because it solves a coupled inverse problem, but the cost trend remains structured and can be evaluated directly against the simpler baselines.

## 4. Discussion

### 4.1. What Information Is Newly Exploited

VACWT is therefore interpreted here as a numerical proof of concept rather than as an experimentally validated weld inspection procedure. Within the declared benchmark, the evidence supports a narrower claim: late scattered-wave observables provide additional constraints on volumetric defect support. The new information enters at the measurement level. Rather than treating the late window as incoherent background, the method extracts stretching, decorrelation, and energy-ratio measurements and uses them alongside first-arrival travel time perturbation.

This distinction matters because the welded background is itself a distributed scatterer. A first-arrival method can improve its delay law and still inflate support when weak scattered energy is projected into the image volume. The present formulation assigns different physical roles to different observables. Smooth delay is represented by δs, scattering redistribution by δη, and compact defect support by χ. The interference case demonstrates why this separation is useful: the recovered body contracts toward the region supported simultaneously by travel time, stretching, decorrelation, and energy information.

### 4.2. Relation to TFM, RTM, and FWI

TFM is computationally efficient and useful when the propagation model is sufficiently accurate. Corrected TFM improves the delay law by using the known heterogeneous background, but it still forms a focal-amplitude volume. RTM uses a fuller wavefield and handles diffraction and finite-aperture effects more naturally, but the imaging condition does not by itself separate smooth background variation from compact defect occupancy. FWI is more general because it can update material parameters directly. That generality comes at a cost: it requires stronger prior information, repeated forward and adjoint simulations, and careful initialization to avoid local minima.

VACWT does not replace these methods. It is a middle route designed for the specific physical question posed in this paper. It uses a limited set of measurements extracted from the waveform rather than the full time series, and it estimates three effective volumes rather than a full elastic tensor. This reduces the number of unknowns while retaining late-window information that is absent from first-arrival-dominated imaging.

### 4.3. Scope and Limitations

The present study has four explicit limits. First, it is entirely numerical. The benchmark shows what can be achieved under a controlled parameterized welded-solid model, not what is guaranteed for experimental weld specimens. Second, the material fields are effective volumetric fields, not measurements of actual weld metallurgy. Third, the inversion recovers effective slowness, scattering strength, and defect occupancy; it does not recover a unique microstructural model or a full anisotropic stiffness tensor. Fourth, all quantitative conclusions depend on the selected frequency band, aperture, scan geometry, defect family, noise level, and thresholding rule.

These limits are important. The correct interpretation is that late scattered-wave observables improve volumetric defect-support recovery within the declared numerical benchmark. The method remains unproven for real weld noise, couplant variability, probe positioning errors beyond the tested range, anisotropic grain scattering, surface roughness, and imperfect knowledge of weld geometry. The next necessary step is experimental verification on welded steel plates with known artificial and natural defects.

## 5. Conclusions

This study developed and tested a three-dimensional VACWT formulation for a controlled numerical welded-solid benchmark. The main conclusions are as follows.

(1) The opposing parallel-scan layout can be organized as a virtual array in midpoint–angle–depth space. In the present benchmark, this organization provides a clear way to evaluate volumetric coverage from line-scan data.
(2) First-arrival and late-window measurements carry different physical information. Travel time perturbation primarily constrains smooth path-integrated delay, whereas stretching, decorrelation, and energy ratio constrain late scattered-wave redistribution around the defected region.
(3) Under the specified simulation conditions and the fixed defect-free thresholding rule, VACWT recovered more compact support than DAS-TFM, corrected TFM, and RTM. The largest difference occurred in the interference case, where first-arrival-dominated imaging produced broad non-true volume, whereas the coupled observable formulation preserved a more localized support.
(4) The results should be interpreted as a numerical proof of concept rather than general practical validation. Future work should test the method on experimental phased-array data from welded steel specimens with known defects, quantify the influence of couplant variability and surface condition, and extend the benchmark to measured anisotropic weld maps and multiple inspection frequencies.

## Figures and Tables

**Table 1:** Fixed numerical settings used in the three-dimensional benchmark.

Item	Symbol	Value	Role in the Workflow
Forward domain	$L_{x}\times L_{y}\times L_{z}$	72 × 24 × 120 mm	Three-dimensional welded solid used in the forward calculation
Reconstruction domain	Ω	48 × 20 × 96 mm	Region used for inversion and all reported metrics
Forward grid spacing	Δx=Δy=Δz	0.20 mm	Wavefield simulation grid
Inverse grid spacing	Δx=Δy=Δz	0.25 mm	Reconstruction grid
Probe offsets	a,b	18 mm, 18 mm	Opposing trajectories on both weld sides
Scan samples	$N_{A},N_{B}$	41, 161	6601 cross-weld records
Step sizes	$\Delta z_{A},\Delta z_{B}$	2.0 mm, 0.5 mm	Midpoint-angle density of the virtual aperture
Excitation	$f_{0}$	5 MHz	Millimetre-scale weld defect interrogation in the present plate thickness
Record length and sampling	$T,f_{s}$	110 microseconds, 100 MHz	Ballistic and coda-window analysis
Late window	$t_{c1},t_{c2}$	25 microseconds, 70 microseconds	Interval used for stretching, decorrelation, and energy ratio
Absorbing boundary	-	2.4 mm	Boundary reflection suppression
Parent reference	$c_{L},c_{S},\rho$	5900 m/s, 3230 m/s, 7850 kg/m^3^	Generic carbon steel reference, not a named steel grade

**Table 2:** Inversion weights, regularization parameters, and stopping criteria.

Quantity	Value Used in Main Benchmark	Selection Rule
Travel time standard deviation	$\sigma_{\tau }=0.03$ ms	Estimated from defect-free repeated synthetic traces
Stretching standard deviation	$\sigma_{\epsilon }=0.12\times 10^{-3}$	Estimated from defect-free late-window correlation
Decorrelation standard deviation	$\sigma_{D}=0.025$	Estimated from defect-free late-window residuals
Energy ratio standard deviation	$\sigma_{R}=0.08$	Estimated from defect-free late-window energy variation
Data weights	$w_{k}=1/\sigma_{k}$	Applied separately to δτ,ϵ,D,R
Slowness smoothness	$\lambda_{s}=3.0\times 10^{-3}$	Selected from an L-curve on the cylindrical-void case and fixed afterwards
Scattering smoothness	$\lambda_{\eta }=1.0\times 10^{-3}$	Same fixed value for all defect families
Defect-support TV weight	$\lambda_{\chi }=5.0\times 10^{-4}$	Fixed after defect-free calibration
TV differentiability parameter	$10^{-6}$	Prevents singular IRLS weights
Gauss–Newton iterations	8 maximum	Stopped earlier if relative objective decrease was below $10^{-4}$
IRLS iterations per outer step	5 maximum	Applied to the TV term
Linear solver tolerance	$10^{-5}$ relative residual	Conjugate-gradient inner solve
Maximum CG iterations	80	Fixed for all methods using iterative solves
Positivity constraint	χ≥0	Enforced by projection after each support update

**Table 3:** Support extraction and volumetric metric definitions.

Step or Metric	Definition	Unit
Normalized volume	Percentile-normalized image or occupancy volume inside Ω	-
Threshold	Fixed from the defect-free benchmark for each method; not tuned per defect	-
Small component removal	Components smaller than six inverse-grid voxels removed for all methods	voxels
Centroid error	Euclidean distance between true and recovered support centroids	mm
Volumetric IoU	Intersection volume divided by union volume between recovered and true supports	-
False-positive volume fraction	Recovered support outside the true support divided by recovered support volume, multiplied by 100	%
Image-domain SNR gain	Contrast gain relative to DAS-TFM in the same benchmark	dB

## Data Availability

The original contributions presented in this study are included in the article. Further inquiries can be directed to the corresponding author.
